# Endovascular management of bilateral renal angiomyolipoma in a perimenopausal woman

**DOI:** 10.1590/1677-5449.202000672

**Published:** 2023-08-28

**Authors:** Ganesh Govinda Gowda, Maureen Tigga, Ravikumar Banavase Ramesh

**Affiliations:** 1 JSS Medical College & Hospital, Mysore, Karnataka, India.

**Keywords:** angiomyolipoma, kidney, angiography, angiomiolipoma, rim, angiografia

## Abstract

Renal angiomyolipoma is a benign and progressive tumor consisting of smooth muscle, fat, and vascular elements and is commonly associated with the tuberous sclerosis complex. Bilateral occurrence is rare and recent evidence suggest strong tumor positivity to female hormones with enlargement during pregnancy and oral contraceptive therapy. Treatment varies from a minimally invasive approach with selective embolization of the renal artery to partial nephrectomy or sometimes even radical nephrectomy. Presented here is a case of bilateral renal angiomyolipoma in 50-year-old lady who was successfully treated with super-selective embolization.

## Resumo

O angiomiolipoma renal é um tumor benigno e progressivo composto por músculo liso, gordura e elementos vasculares, comumente associado ao complexo da esclerose tuberosa. Sua ocorrência bilateral é rara, e evidências recentes sugerem forte positividade do tumor para hormônios femininos, com aumento do tamanho durante a gravidez e uso de contraceptivos orais. O tratamento varia desde uma abordagem minimamente invasiva de embolização seletiva da artéria renal até nefrectomia parcial ou, às vezes, nefrectomia radical. Apresentamos um caso de angiomiolipoma renal bilateral em uma mulher de 50 anos que passou por tratamento bem-sucedido com embolização superseletiva.

## INTRODUCTION

The incidence of renal angiomyolipma (AML) is reported to be 0.2-0.6% in the general population, reflecting the rarity of its occurrence.^1^ It is the most frequent benign mesenchymal tumor of the kidney, comprising abnormal thick-walled blood vessels, spindle and epithelioid smooth muscle cells, and adipose tissue and it has been confirmed that it belongs to the perivascular epithelioid cell tumor family.^2^ These tumors usually arise in the renal cortex, but on extremely rare occasions they have been reported to originate from the renal sinus and cause compression of the renal pelvis.^3^ Two well-known types have been distinguished, sporadic AML and AML associated with tuberous sclerosis complex (TSC), which is an autosomal dominant disease that affects several organs, e.g. brain, skin, eyes, heart, kidney, and lungs.^4^ Most isolated AMLs are found through incidental imaging and sporadic variants are more frequent and are typically diagnosed in patients with a mean age of 43 years.^4^

Typical renal AML can be accurately diagnosed with imaging modalities like computed tomography and magnetic resonance imaging. Presence of intralesional fat is the hallmark feature in all modalities.^5^ Radiologically, AMLs have been classified as fat-rich, fat-poor, or fat-invisible based on the amount of adipose tissue detected on imaging.^5^ Patients are usually asymptomatic, especially when the lesion is less than 4 cm, while lesions greater than 4 cm in diameter present with lumbar pain, anemia and hematuria.^6^

Retroperitoneal hemorrhage or bleeding into the renal collecting system are the major complications which may jeopardize a patient’s life.^7^ Therapeutic strategies that have been described include conservative selective embolization of the renal artery, nephrectomy for severe cases, and even medical approaches with agents like sirolimus.^8^ AML has a strong predilection for the female population, being four times more frequent in women than in men.^9^ Recent evidence suggests strong tumor positivity to female hormones with enlargement during pregnancy and oral contraceptive therapy.^10^ Bilateral occurrence of AML is also extremely rare and infrequently described in literature. Presented here is a case of bilateral renal angiomyolipoma in a 50-year-old patient with a history of menorrhagia treated with hormonal contraceptives who was managed by selective embolization of the bilateral renal arteries.

## CASE REPORT

**Figure 1:**
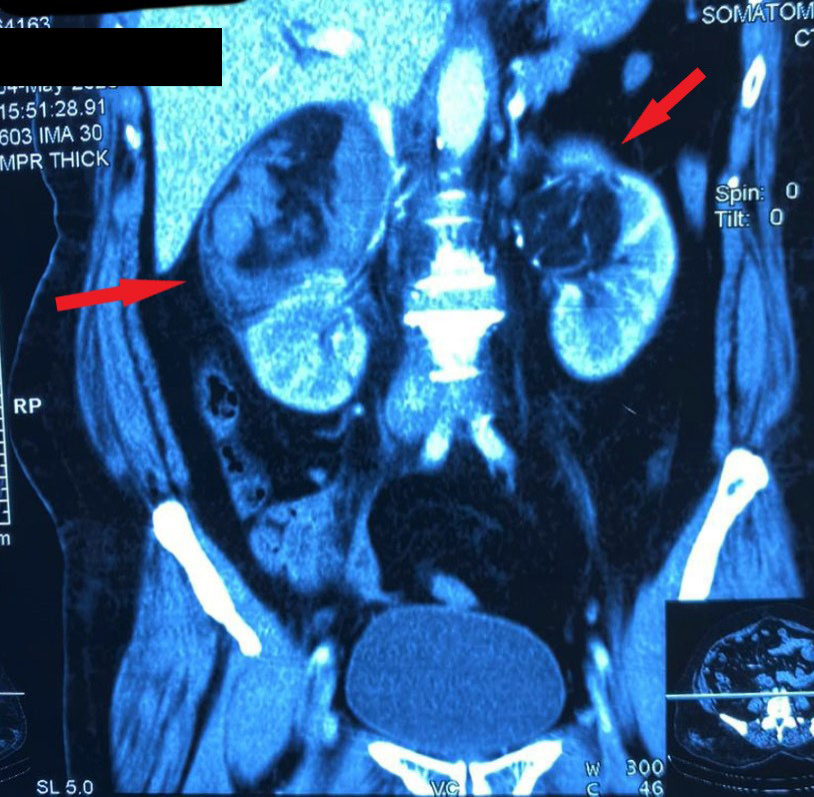
Computed tomography image of bilateral renal angiomyolipoma.

A 50-year-old perimenopausal patient presented to the outpatient department with abdominal pain, lower backache, and dysuria. She was multiparous with a history of metromenorrhagia and had been taking oral contraceptive pills on and off to control her symptoms. Her gynecological examination revealed mild white discharge per vagina with no structural abnormalities and she was therefore treated with local antibiotics and sent for an abdominopelvic ultrasonography. Ultrasound revealed a bulky uterus with normal adnexa and bilateral hyperechoic renal lesions which measured 7 cm on the right side and 4.5 cm on the left side. A contrast enhanced computed tomography (CECT) of the abdomen reported well-defined lesions of 9 x 5 cm on right side and 5 x 4.5 cm on left side with fat density within the upper and interpolar regions of both kidneys suggestive of bilateral fat rich AML (Figure 1).

**Figure 2:**
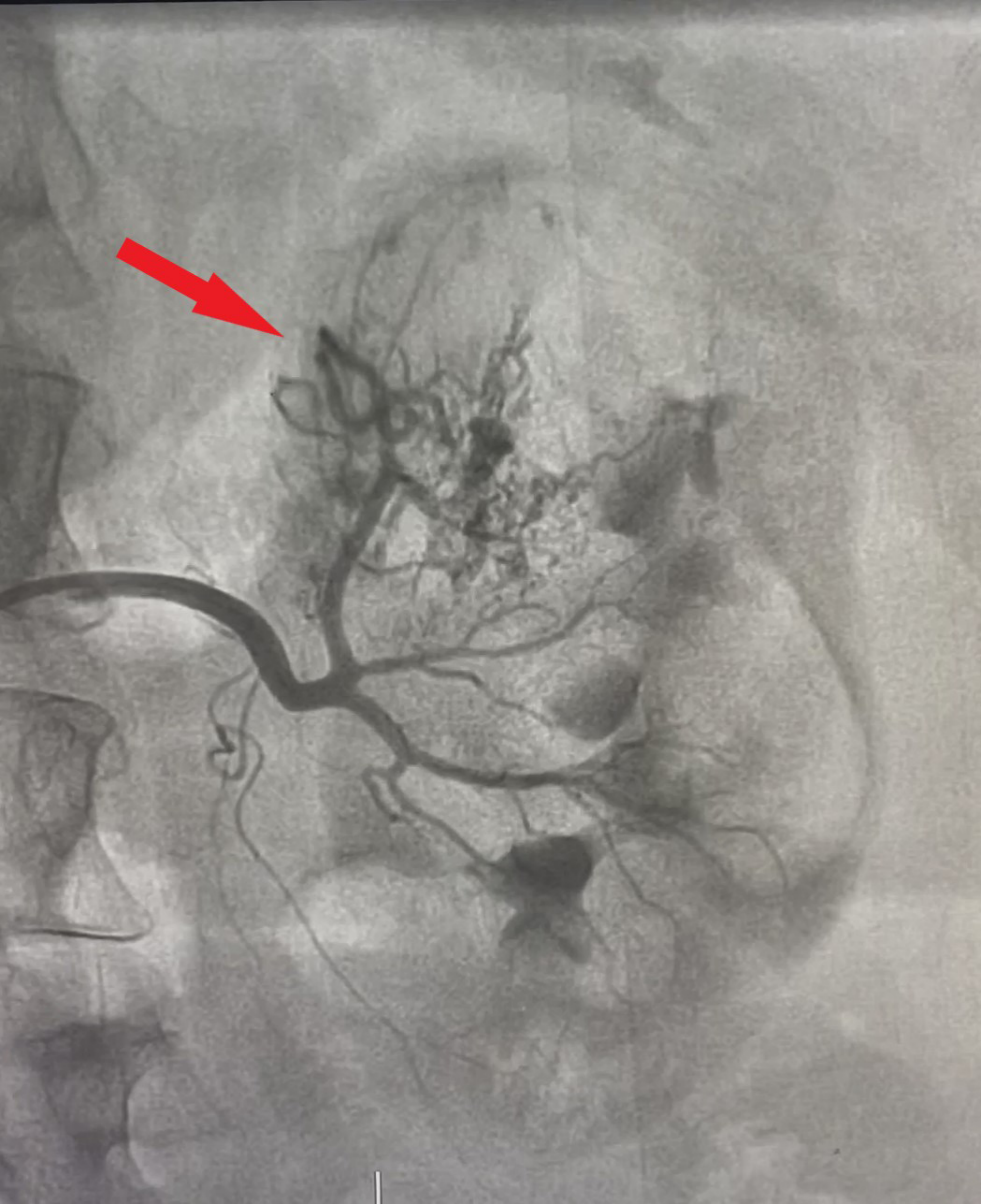
Angiogram of left renal angiomyolipoma showing feeding vessels from the middle polar artery forming a contrast blush.

**Figure 3:**
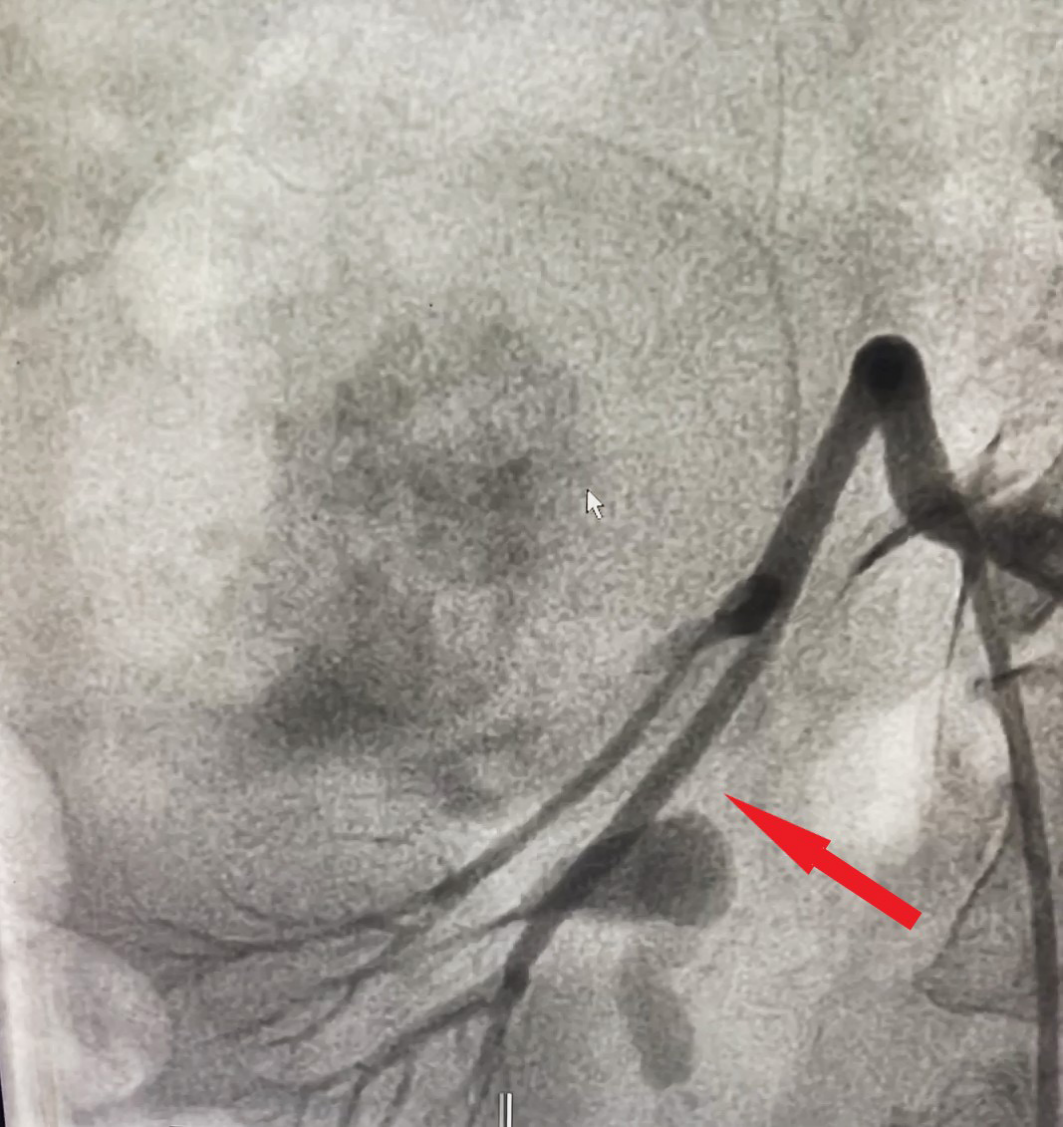
Post-embolization angiogram of left renal angiomyolipoma showing absent contrast blush.

**Figure 4:**
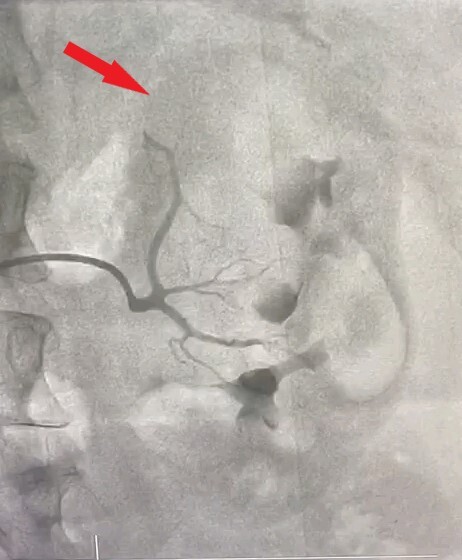
Angiogram of right renal angiomyolipoma showing feeding vessels arising from the upper polar artery.

**Figure 5:**
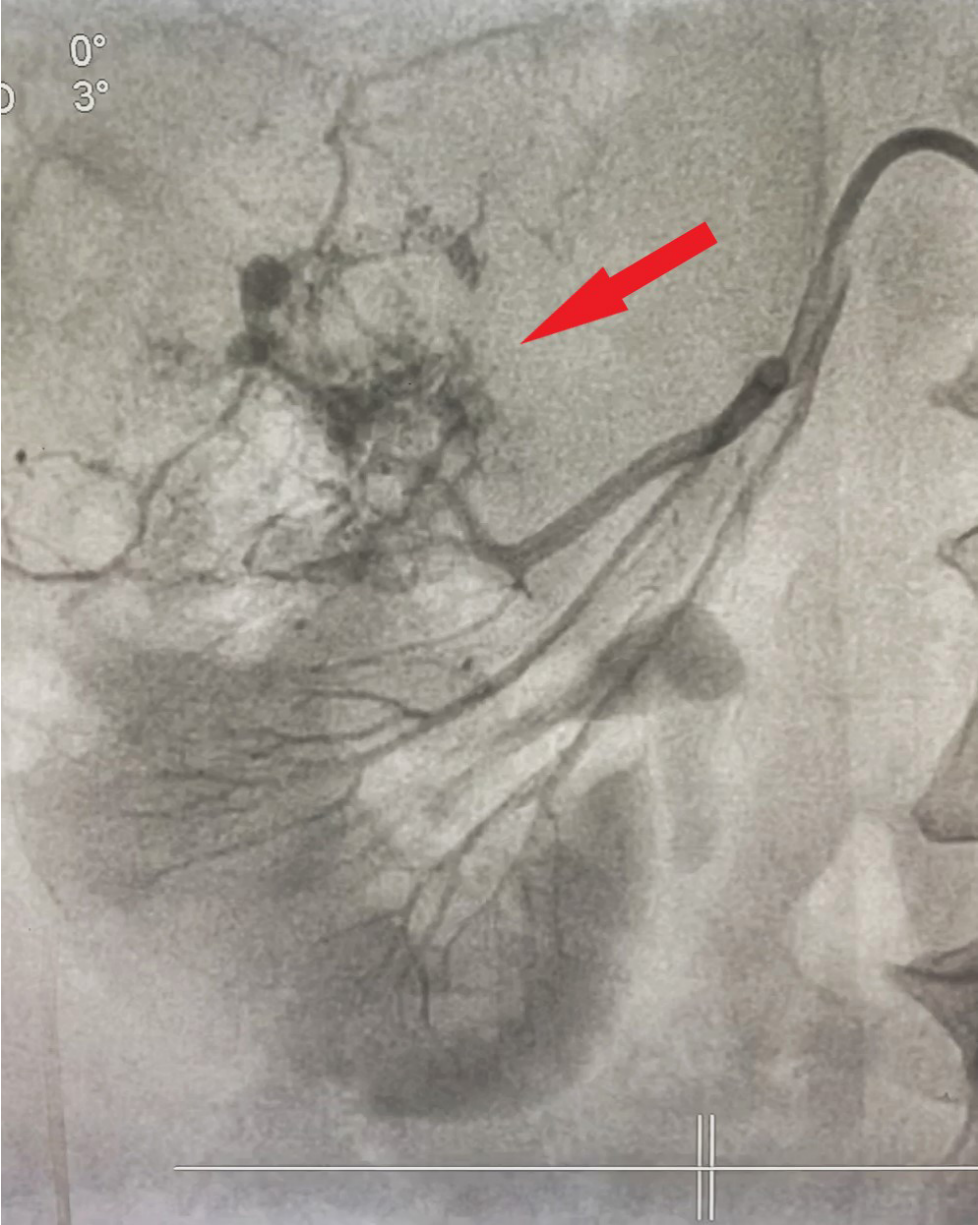
Post embolization angiogram of right renal angiomyolipoma showing the embolized upper polar artery and the preserved middle and lower polar arteries.

As CECT has high accuracy for detecting renal AML, we proceeded to treatment without performing biopsy. The lesions were greater than 4 cm in size, reflecting preponderance for rupture, and involved both kidneys, so the patient was posted for super-selective angioembolization (SAE). The right femoral artery was punctured under local anesthesia and a 6F hemostatic sheath was placed. The left renal artery was catheterized using a 5F JR catheter and angiograms were taken in multiple planes, revealing feeding vessels from the middle polar artery forming a contrast blush (Figure 2). The upper and lower polar arteries and their branches were normal. The left middle polar artery was then super-selectively catheterized using a microcatheter advanced as close to the lesion as possible. After confirming the position, polyvinyl alcohol (PVA) particles (250-350 microns) were injected. A post-embolization angiogram revealed absent contrast blush (Figure 3). The left main renal artery and its upper and lower polar branches were all preserved. Thereafter, the right renal artery was catheterized and angiograms were taken in multiple planes, revealing feeding vessels arising from the upper polar artery (Figure 4). A similar procedure was repeated on the right side to embolize the upper polar artery while preserving the middle and lower polar arteries (Figure 5). Post procedure, the patient’s renal function remained unaltered. Her pre-procedural renal function test showed creatinine level of 0.7 mg/dL while her post procedure creatinine level was 0.8 mg/dL. Her recovery period was uneventful and she was discharged on the second postoperative day.

## DISCUSSION

Renal AML is a benign tumor and indications for treatment include intractable pain, hematuria, spontaneous rupture with hemodynamic instability, large tumors, and imaging suggestive of malignant lesions.^11^ Oesterling et al.^11^ suggested that tumors > 4 cm are usually symptomatic with a tendency to bleed and therefore require either selective arterial embolization (SAE) or surgical treatments such as partial nephrectomy, enucleation, or wedge resection, whereas tumors < 4 cm should be followed up with yearly CT scans or ultrasonography.^11^

SAE of the renal artery is safe and effective for symptomatic and large-sized AMLs, which have shown a mean reduction in size by about 43%.^12^ However, in a small number of cases they have been reported to have enlarged due to an increased nonvascular component. In such cases, it is recommended to repeat angiography, reconfirm the diagnosis of AML, and re-treat the lesion by SAE where appropriate. In a study by Ramon et al.,^12^ patients initially treated with SAE were followed up for a period of 4.8 years, during which no symptoms such as pain or bleeding occurred.^12^ However, repeat embolization was needed in about 37% of cases due to neo-angiogenesis or re-canalization of treated vessels.^12^ These authors also reported significantly lower rates of post-embolization syndrome (i.e. fever and flank pain) and no deaths or changes to kidney function were reported in relation to SAE.^12^ They concluded that SAE is minimally invasive and associated with optimal preservation of renal function.^12^ In our patient, the tumor size was greater than 4 cm in both kidneys, with a high predilection for rupture, and SAE achieved good outcomes.^11^ The patient’s renal function remained unaltered, sparing both kidneys, and she recovered well. Noteworthy features of our case were the occurrence of bilateral renal AML, which is extremely rare, and the associated history of hormonal contraceptive use, which other authors have reported as increasing the size of AML.

## CONCLUSION

Bilateral renal angiomyolipoma is a less commonly encountered entity in clinical practice. Treatment with SAE has the advantage of being minimally invasive and providing promising results in patients in whom both kidneys are affected. SAE proves to be an effective and minimally invasive procedure allowing preservation of renal function.
